# Canadian physiotherapists' views on certification, specialisation, extended role practice, and entry-level training in rheumatology

**DOI:** 10.1186/1472-6963-9-88

**Published:** 2009-06-02

**Authors:** Linda C Li, Marie D Westby, Evelyn Sutton, Marlene Thompson, Eric C Sayre, Lynn Casimiro

**Affiliations:** 1 Department of Physical Therapy, University of British Columbia, Vancouver, Canada; 2 Arthritis Research Centre of Canada, Vancouver, Canada; 3 Mary Pack Arthritis Program, Vancouver General Hospital, University of British Columbia, Vancouver, Canada; 4 Department of Medicine, Dalhousie University, Halifax, Canada; 5 Nova Scotia Arthritis Centre, Queen Elizabeth II Health Sciences Centre, Halifax, Canada; 6 St. Joseph's Hospital, London, Canada; 7 Department of Statistics and Actuarial Science, Simon Fraser University, Vancouver, Canada; 8 Academic Health Council, University of Ottawa, Ottawa, Canada

## Abstract

**Background:**

Since the last decade there has been a gradual change of boundaries of health professions in providing arthritis care. In Canada, some facilities have begun to adopt new arthritis care models, some of which involve physiotherapists (PT) working in extended roles. However, little is known about PTs' interests in these new roles. The primary objective of this survey was to determine the interests among orthopaedic physiotherapists (PTs) in being a certified arthritis therapist, a PT specialized in arthritis, or an extended scope practitioner in rheumatology, and to explore the associated factors, including the coverage of arthritis content in the entry-level physiotherapy training.

**Methods:**

Six hundred PTs practicing in orthopaedics in Canada were randomly selected to receive a postal survey. The questionnaire covered areas related to clinical practice, perceptions of rheumatology training received, and attitudes toward PT roles in arthritis care. Logistic regression models were developed to explore the associations between PTs' interests in pursuing each of the three extended scope practice designations and the personal/professional/attitudinal variables.

**Results:**

We received 286 questionnaires (response rate = 47.7%); 258 contained usable data. The average length of time in practice was 15.4 years (SD = 10.4). About 1 in 4 PTs agreed that they were interested in assuming advanced practice roles (being a certified arthritis therapist = 28.9%, being a PT specialized in rheumatology = 23.3%, being a PT practitioner = 20.9%). Having a caseload of ≥ 40% in arthritis, having a positive attitude toward advanced practice roles in arthritis care and toward the formal credentialing process, and recognizing the difference between certification and specialisation were associated with an interest in pursing advanced practice roles.

**Conclusion:**

Orthopaedic PTs in Canada indicated a fair level of interest in pursuing certification, specialisation and extended scope practice roles in arthritis care. Future research should focus on the effectiveness and cost-effectiveness of the emerging health service delivery models involving certified, specialized or extended scope practice PTs in the management of arthritis.

## Background

The delivery of health services to people with arthritis is undergoing transformation in Canada. Since the last decade there has been a gradual change of boundaries of health professions in providing arthritis care [1,2]. The trend is particularly evident in nursing and rehabilitation professions where clinicians begin to assume roles that are outside of their usual scope of practice while working in a multidisciplinary teams [3].

Physiotherapists (PTs) are in a unique position to expand their roles in arthritis care because of the focus in the musculoskeletal system and orthopaedic conditions during their entry-level training. In Canada, some PTs and occupational therapists work as multi-skilled primary therapists and provide services that cross the traditional physiotherapy/occupational therapy boundary [4,5]. Training workshops are available, although not mandatory, for arthritis primary therapists [6]. To our knowledge, fewer than 1% of PTs have also received additional training to perform more advanced tasks such as conducting musculoskeletal examinations, ordering investigative tests, and providing referrals to specialists or other health professionals [7-10]. Different titles have been given to these skilled health professionals, including extended scope practitioners [11], and advanced practitioners [10]. According to the Chartered Society of Physiotherapy in the UK, extended scope practitioners are defined as PTs who work beyond the recognized scope of practice in non-traditional roles [11].

A few reasons contribute to the expansion of roles of PTs in arthritis care, including the shortage of local specialists and facilities [12], and the evidence that supports early diagnosis and treatment [13-15]. A recent systematic review also cited improving patient outcome and ensuring the safety of patients and therapists as the drivers for the development of extended scope physiotherapy practitioners [16]. In Canada the most significant driving force is perhaps the long waiting time for specialist consultations and surgery [17,18]. Between 2000 and 2001, fewer than 50% of patients in Ontario received their joint replacement surgery within six months [19], which was the maximum waiting time identified by several expert panels [20,21]. This situation was expected to deteriorate further due to the critical shortage of orthopaedic surgeons [12]. Since surgeons can potentially spend more time performing surgery if their consultation time focuses mainly on patients who are surgical candidates, some facilities have developed extended scope PT positions to screen orthopaedic referrals and provide treatments for those who do not require surgery [10,22]. Some facilities also train and employ extended scope PTs to monitor patients with stable rheumatological disease [9]. In addition to addressing gaps in arthritis care, these initiatives also serve to provide opportunities for career advancement for PTs practicing in orthopaedics.

In a previous literature review on the effectiveness of treatment provided by PTs in their traditional roles to patients with rheumatoid arthritis (RA), we found benefits in the patient's physical function, self-efficacy and disease-specific knowledge, especially when it was delivered by rheumatology-trained therapists [23]. Several studies have also examined the effectiveness of extended scope PT practitioners in arthritis care. Research from the UK has shown that extended scope PTs can competently triage cases referred for specialist consultations and reduce orthopaedic and rheumatology waiting lists [24-26]. A more recent chart audit study found that about 71% of the orthopaedic consultation referrals made by extended scope PTs from one UK facility were appropriate [27]. To put this in perspective, two previous studies found that 34% to 43% of the orthopaedic referrals made by general practitioners were considered inappropriate or 'avoidable'[28,29] Furthermore, Campos et al. reported that the level of satisfaction of paediatric patients and their parents, after the child received care in a PT practitioner-led arthritis clinic, was as high as those seen at a physician-led clinic[8]

In Canada, there is currently no formal process of certification or specialisation [30] (see Table 1 for the definitions) for PTs who work in non-traditional roles in arthritis care. A few facilities have developed training programs [7] and new extended scope PT positions in rheumatology [8,9] and orthopaedics;[10] however, the development of national standards for training and practice is only at the early stage. A key element to the implementation of extended scope physiotherapy is the interest of PTs in pursuing new roles in arthritis care, which we currently know little about. The absence of this information is a barrier to the planning of resources for education and credentialing across the country.

**Table 1:** Definitions of 'certification', 'specialisation' and 'extended role practitioner' used in this survey

**Certification **is defined as:
*Program and process where a learner completes prescribed training and passes an assessment with a minimum acceptable score*

**Physiotherapy specialisation **is defined as:
*"....the application of advanced clinical competence by a physiotherapist qualified in a defined area of practice within the field of activity recognised as physiotherapy." *[30]

**Extended scope practitioner **is defined as:
....*therapists who are working beyond the recognized scope of practice of the profession of interest in innovative or non-traditional roles* This includes: *"....requesting investigations e.g., blood tests, scans, nerve conduction studies; using the results of investigations to assist clinical diagnosis and appropriate management of patients; listing for surgery and referring to other medical and paramedical professionals." *[11]

The primary objective of this study was to determine the interests of orthopaedic PTs in pursuing certification, specialisation and extended scope practice roles in rheumatology, and to explore the associated factors. In addition, we assessed the coverage of the rheumatology content during the entry-level physiotherapy education from the perspectives of practicing PTs. Findings of this study are particularly useful for health care administrators, regulators and educators to develop training resources and competency standards on extended scope practice in rheumatology.

## Methods

This study was a part of the 2007 *Canadian PT Arthritis Care Survey *that aimed to understand therapists' practices in arthritis care, education needs and views on emerging professional roles. Individuals were eligible if they were PTs who practiced in orthopaedics in Canada. Of the 10 regulatory colleges of the physiotherapy profession, nine agreed to participate and subsequently provided assistance to identify eligible PTs (n = 6,994). One college declined to participate due to its internal policy. We randomly selected 600 eligible PTs to receive the questionnaire using a computer-generated table of random numbers.

### Questionnaire development and administration

The questionnaire was developed by a team consisting of a PT practicing in rheumatology (MT), a rheumatologist (ES), a physiotherapy educator (MDW) and two researchers (LCL, LC), and built on the work of the UK Arthritis Research Campaign (ARC). The ARC project aimed to define the extended roles of PTs, occupational therapists and nurses in rheumatology through a series of surveys and workshops[31] The core clinical competencies for PTs and education needs identified by ARC formed the bases for item generation in the current survey. The questionnaire covered four areas related to clinical practice, knowledge, and attitude toward PT roles in rheumatology: 1.) current practice and roles in assessment and treatment; 2.) therapists' confidence in arthritis management; 3.) content of rheumatology training; and 4.) general views on certification, specialisation, and extended scope of practice.

The questionnaire was pre-tested for face and content validity with PTs working in orthopaedics (n = 8) or rheumatology (n = 6) in the province of British Columbia. The content was subsequently revised and reviewed by the same volunteers before it was used for the survey. For participants in the province of Québec, we translated the questionnaire into French using a rigorous protocol: 1.) initial translation from English to French by a professional translator, and back-translation into English by a bilingual clinician; 2.) an *ad hoc *committee comprised of a bilingual clinician, an academic and a research team member (LC) examined the forward and backward translated documents and agreed on a provisional French version; 3.) the professional translator translated the provisional version into English; 4.) the *ad hoc *committee compared the back-translated English version with the original questionnaire and revised the wording of the French version; 5.) four bilingual clinicians who were not involved in the process reviewed the documents; two of them completed the French and English questionnaires to determine if the versions were the same, and the other two rated each statement in the French version for clarity; and (6.) LC made the final edits according to the clinicians' comments.

We employed the modified Dillman technique[32,33] in order to elicit the fullest participation in this survey. For the first mailing (March 2007), a covering letter was mailed along with the survey. The letter explained the intent of the study and that the completion and return of the questionnaire indicated the individual's consent to participate in this study. Three weeks later, a reminder postcard was sent to non-respondents. A second and third reminder letter and another copy of the survey were sent to the remaining non-respondents six weeks and eight weeks after the initial mailing. To ensure data quality, we performed double data entry on all questionnaires and then randomly selected 10% of the questionnaires to check for data entry error.

### Variables

For the dependent variables, PTs were asked to indicate on a five-point scale if they agreed that they were interested in becoming: 1.) a certified arthritis therapist; 2.) a PT specializing in rheumatology; and 3.) a PT practitioner in rheumatology. Table 1 presents the definitions of 'certification', 'specialisation' and 'extended scope practitioner' as presented in the questionnaire.

Independent variables included: 1.) coverage of rheumatology in entry-level training; 2.) attitudes toward advanced practice roles; 3.) participation in post-entry-level arthritis courses; 4.) practice characteristics, including current arthritis and joint replacement caseloads, and types of practice settings; and 5.) personal characteristics, including age, sex, and year of graduation from the entry-level training.

Rheumatology entry-level training was assessed by a 26-item scale that addressed the coverage of history taking and pathophysiology (6 items), assessment for three conditions (osteoarthritis (OA), rheumatoid arthritis (RA), and ankylosing spondylitis (AS); 13 items), and treatment (7 items). Participants were asked to rate each item as 'not covered', 'covered, but not adequate', 'adequately covered' or 'can't remember'. The rigor of entry-level training was measured by the total number of areas that were rated as 'adequately covered' (i.e., the score ranged from 0 to 26). Attitudes toward advanced practice in rheumatology were assessed by 15 statements rated on a 5-point Likert scale, ranging from 'strongly agree' to 'strongly disagree'.

Participants' return of the questionnaire was their implied consent to participate in the study. The study protocol was approved by the University of British Columbia Behavioural Research Ethics Board (Application number: B06-0719)

### Statistical analysis

Participant characteristics were assessed using frequencies, or means and standard deviations depending on the measure. We developed logistic regression models to explore the associations between PTs' interests in pursuing each of the three physiotherapy practice designations (strongly agree/agree = 1; strongly disagree/disagree/not sure = 0) and the following independent variables: 1.) sex; 2.) age (≥ age 35 vs. <age 35); 3.) number of years since graduation from entry-level training (≤ 10 years vs. >10 years); 4.) participation in post-entry-level arthritis courses (yes/no); 5.) types of practice setting (multidisciplinary team vs. other); 6.) arthritis caseload (≥ 40% of patients with OA or RA vs. <40%); 7.) surgical caseload (≥ 40% of total joint replacement cases vs. <40%); 8.) rating of entry-level rheumatology content (scored ≥ 13 out of 26 in content rigor as 'adequately covered' vs. <13); and 9.) attitudes toward potential roles of advanced practitioners (strongly agree/agree, not sure, disagree/strongly disagree).

Initially, univariate logistic regression models were fitted on each independent variable versus each of the three dependent variables. Independent variables that passed the pre-screening at α = 0.10 were then entered simultaneously into a backwards elimination logistic regression procedure at α = 0.05. Multi-categorical questions were assessed via the lowest parameter p-value. All variables in the final models were significant at α = 0.05. We assessed model fit using max-rescaled R^2 ^and the c statistic (area under the ROC curve).

## Results

### Participant characteristics

We received 286 of 600 questionnaires (response rate = 47.7%). Of those, 28 participants did not practice clinically in the past year, leaving 258 for the analysis (Table 2). Almost half of these participants were from the province of Ontario (n = 125; 48.4%), 72.5% were females, and 36.4% were under 35 years old. The vast majority (94.2%) received at least a baccalaureate degree or a diploma for the entry-level physiotherapy training. The average length of time in practice was 15.4 years (SD = 10.4) and 60.1% graduated more than 10 years ago. Sixty-three participants (24.4%) completed at least one post-entry-level course on arthritis. About 45% of participants practiced in a multidisciplinary setting. Twenty-eight percent of PTs had a significant arthritis caseload (defined as ≥ 40% of patients with OA or RA in the total caseload in a typical week) and 14.3% had a significant joint replacement caseload.

**Table 2:** Demographic and practice characteristics (n = 258)

	**f(%)**
Region	
Eastern Canada – New Brunswick, Nova Scotia, Newfoundland, Prince Edward Island	22 (8.5)
Quebec	72 (27.9)
Ontario	125 (48.4)
Western Canada – Manitoba, Saskatchewan, Alberta	39 (15.1)
Sex	
Female	187 (72.5)
Male	71 (27.5)
Age	
20 – 34	94 (36.4)
35 – 49	114 (44.2)
50 – 64	46 (17.8)
≥ 65	3 (1.2)
*Missing*	*1 (0.4)*
Education*	
Entry-level PT – Baccalaureate degree or Diploma	243 (94.2)
Entry-level PT – Clinical Master degree	8 (3.1)
Master's degree – thesis-based	15 (5.8)
PhD	0
Years since graduation from entry-level training	
≤ 10 years	100 (38.8)
> 10 years	155 (60.1)
*Missing*	*3 (1.2)*
Years in practice *(SD)*	15.4 *(10.4)*
Completed 1 or more post-entry-level courses on arthritis	63 (24.4)
Type of practice*	
Multidisciplinary team	116 (45)
Group practice with only physiotherapists	58 (22.5)
Solo practice	42 (16.3)
Home care	30 (11.6)
The Arthritis Society (Ontario only)	4 (1.6)
Caseload*	
PTs with ≥ 40% OA or RA cases in a typical week	71 (27.5)
PTs with ≥ 40% joint replacement rehabilitation cases in a typical week	37 (14.3)

* Individuals may report more than one category

f = Frequency

SD = Standard deviation

OA = Osteoarthritis

RA = Rheumatoid arthritis

The majority of participants (>50%) reported that the following topics were adequately covered during their entry-level training (Table 3): history taking (OA: 80.3%; RA: 68.1%; AS: 49.2%) & pathophysiology (OA: 88.2%; RA: 81.1%; AS: 62.8%), exercise prescription (OA: 77.5%; RA: 62.8%: AS: 50.8%), pre/post-surgical care (74.7%), assessment and prescription of mobility aids (68.0%), damaged joint assessment (53.0%), and self-management strategies (51.2%). However, most of the topics on patient assessment were poorly covered or not covered. Also, less than 20% of PTs said that the coverage of community and professional resources for arthritis management was adequate. One hundred and five participants had an entry-level rheumatology content rigor score of 13 or higher, indicating only 41% of PTs rated at least half of the arthritis content adequately covered.

**Table 3:** Coverage of arthritis content at entry-level level training

	N*	**Adequately covered (%)**	**Covered, but not adequate (%)**	**Not Covered (%)**	**Can't remember (%)**
**History taking & Pathophysiology**					
History taking for OA	254	202 (80.3)	25 (9.8)	2 (0.8)	23 (9.1)
History taking for RA	254	173 (68.1)	50 (19.7)	3 (1.2)	28 (11.0)
History taking for AS	254	125 (49.2)	73 (28.7)	11 (4.3)	45 (17.7)
Pathophysiology of OA	254	224 (88.2)	16 (6.3)	1 (0.4)	13 (5.1)
Pathophysiology of RA	254	206 (81.1)	32 (12.6)	1 (0.4)	15 (5.9)
Pathophysiology of AS	253	159 (62.8)	62 (24.5)	3 (1.2)	29 (11.5)
**Assessment**					
Active joint count	254	108 (42.5)	43 (16.9)	62 (24.4)	41 (16.1)
Damaged joint assessment	249	132 (53.0)	67 (26.9)	20 (8.0)	30 (12.0)
Back assessment for AS	253	88 (34.8)	92 (36.4)	28 (11.1)	45 (17.8)
Assessment for Juvenile Inflammatory Arthritis	253	34 (13.4)	102 (40.3)	54 (21.3)	63 (24.9)
Assessment of psychosocial needs	253	43 (17.0)	105 (41.5)	61 (24.1)	44 (17.4)
Read joint x-rays	253	59 (23.3)	110 (43.5)	64 (25.3)	20 (7.9)
Read blood work results	253	35 (13.8)	85 (33.6)	105 (41.5)	28 (11.1)
Use of disease-specific outcome measures	251	44 (17.5)	84 (33.5)	80 (31.9)	43 (17.1)
Assessment/prescription of mobility aids	253	172 (68.0)	55 (21.7)	14 (5.5)	12 (4.7)
Assessment/prescription of adaptive aids	250	98 (39.2)	99 (39.6)	33 (13.2)	20 (8.0)
Assessment/prescription of hand orthoses	253	23 (9.1)	94 (37.2)	108 (42.7)	28 (11.1)
Assessment/prescription of knee braces	254	45 (17.7)	105 (41.3)	81 (31.9)	23 (9.1)
Assessment/prescription of foot orthoses	253	35 (13.8)	107 (42.3)	85 (33.6)	26 (10.3)
**Treatment**					
Exercise prescription for patients with OA	253	196 (77.5)	40 (15.8)	3 (1.2)	14 (5.5)
Exercise prescription for patients with RA	253	159 (62.8)	66 (26.1)	6 (2.4)	22 (8.7)
Exercise prescription for patients with AS	252	128 (50.8)	75 (29.8)	14 (5.6)	35 (13.9)
Pre/post-total joint replacement care	253	189 (74.7)	30 (11.9)	17 (6.7)	17 (6.7)
Self-management education	254	130 (51.2)	75 (29.5)	20 (7.9)	29 (11.4)
Availability of community resources for people with arthritis	254	48 (18.9)	78 (30.7)	72 (28.3)	56 (22.0)
Availability of professional resources for arthritis management	253	42 (16.6)	88 (34.8)	71 (28.1)	52 (20.6)

* N = number of participants provided a response

OA = osteoarthritis; RA = rheumatoid arthritis; AS = ankylosing spondylitis

### PTs' interests in certification, specialisation and extended role practice in rheumatology

28.9% of PTs strongly agreed/agreed that they were interested in being a certified arthritis therapist, 23.3% being a PT specializing in rheumatology, and 20.9% being a PT practitioner in rheumatology (Table 4). The vast majority of participants felt that PTs could play an important role in screening and early identification of arthritis (96.1%), and that they should be trained to triage patients for rheumatologists (65.4%). However, fewer agreed that PTs with advanced arthritis training should triage all patients referred to orthopaedic surgical consultation (44.1%), adjust medications and order investigative tests under physician supervision (46.0%), or perform injections under physician supervision (34.1%). Most of the participants strongly agreed/agreed that certification or specialisation could help to raise the profile of PTs practicing in the arthritis field (71.4% and 83.1%, respectively), but only 18.4% felt that the current salary structure differentiated the different levels of training and credentials.

**Table 4:** Physiotherapists' views toward certification, specialisation, and extended scope practice roles

	N*	**Strongly agree/agree (%)**	**Not sure (%)**	**Strongly disagree/Disagree (%)**
**I would be interested in being a certified arthritis therapist**	**253**	**73 (28.9)**	**75 (29.6)**	**105 (41.5)**
**I would be interested in being a PT specializing in rheumatology**	**253**	**59 (23.3)**	**65 (25.7)**	**129 (51.0)**
**I would be interested in being a PT practitioner in rheumatology**	**253**	**53 (20.9)**	**57 (22.5)**	**143 (56.5)**
All PTs working in rheumatology should be certified as "Arthritis/Rheumatology Therapists"	253	89 (35.2)	56 (22.1)	108 (42.7)
The certification process would not improve the care for people with arthritis	255	28 (11.0)	62 (24.3)	165 (64.7)
A Certified Arthritis Therapist should be able to interpret findings from clinical research studies	255	222 (87.1)	21 (8.2)	12 (4.7)
The current salary structure differentiates entry-level trained PTs, certified PTs, & PT Practitioners	255	47 (18.4)	87 (34.1)	121 (47.5)
I see no difference between "certification" and "specialisation" in physiotherapy	252	53 (21.0)	43 (17.1)	156 (61.9)
Certification can help raise the profile of PTs practicing in the arthritis field	255	182 (71.4)	48 (18.8)	25 (9.8)
Specialisation can help raise the profile of PTs practicing in the arthritis field	254	211 (83.1)	29 (11.4)	14 (5.5)
It should be mandatory for PTs specialized in arthritis to participate in research activities	252	51 (20.2)	61 (24.2)	140 (55.6)
In Canada, less than 20% of PTs in arthritis rehabilitation will be interested in getting certified	254	47 (18.5)	160 (63.0)	47 (18.5)
In Canada, less than 20% of PTs in arthritis rehabilitation will be interested in being a specialist	253	53 (20.9)	155 (61.3)	45 (17.8)
PTs can play an important role in screening and the early identification of arthritis	254	244 (96.1)	6 (2.4)	4 (1.6)
Orthopaedic PTs should be trained to triage patients for rheumatologists	254	166 (65.4)	67 (26.4)	21 (8.3)
All patients referred to see an orthopaedic surgeon for consultation should be first triaged by a PT with advanced arthritis training	254	112 (44.1)	90 (35.4)	52 (20.5)
PT Practitioners should be allowed to adjust medications and order investigative tests under the supervision of a physician	252	116 (46.0)	65 (25.8)	71 (28.2)
PT Practitioners should be allowed to perform injections under the supervision of a physician	252	86 (34.1)	66 (26.2)	100 (39.7)

* N = number of participants provided a response

PT = physiotherapist

Logistic regression models were developed to assess factors associated with PTs' interests in certification, specialisation and extended role practice. There were 258 participants, with between 246 and 251 used in each model after dropping missing data. For comparing two groups, assuming the probability of a dependent variable is 1 in 3 in the reference group, 123 cases per group would yield 76% power to detect a relative risk of 1.5 at α = 0.05.

Table 5 presents the odds ratios (ORs), 95% confidence intervals (CIs) and fit statistics for the final selected logistic models. The model predicting interest in being a certified arthritis therapist retained four statistically significant explanatory variables, including 1.) current OA/RA caseload ≥ 40% (OR = 2.24, 95% CI: 1.17, 4.28); 2.) agreement to "all patients referred to see an orthopaedic surgeon for consultation should be first triaged by a PT with advanced arthritis training" (strongly agree/agree vs. disagree/strongly disagree OR = 3.24, 95% CI: 1.31, 8.01); 3.) agreement to "all PTs working in rheumatology should be certified as arthritis/rheumatology therapists" (strongly agree/agree vs. disagree/strongly disagree OR = 2.22, 95% CI: 1.10, 4.51); and, 4.) agreement to "I see no difference between 'certification' and 'specialisation' in physiotherapy" (strongly agree/agree vs. disagree/strongly disagree OR = 0.34, 95% CI: 0.14, 0.83). All four variables' effects are monotonic (considering the ORs for not sure vs. disagree/strongly disagree) and their associations with the dependent variable are in the expected directions.

**Table 5:** Logistic regression models for physiotherapists' interests in being a certified arthritis therapist, a physiotherapist specialist in rheumatology, or a physiotherapist practitioner in rheumatology

**Variable**	**Odds Ratio (95% CI)**
**Predicting interest in being a Certified Arthritis Therapist **(Max-rescaled R^2 ^= 0.19, c = 0.73)	
Current arthritis caseload	
≥ 40% of patients with OA or RA	2.24 (1.17, 4.28)
<40% of patients with OA or RA	1.00
"All patients referred to see an orthopaedic surgeon for consultation should be first triaged by a PT with advanced arthritis training"	
Strongly agree/agree	3.24 (1.31, 8.01)
Not sure	2.53 (0.99, 6.49)
Disagree/strongly disagree	1.00
"All PTs working in rheumatology should be certified as Arthritis/Rheumatology Therapists"	
Strongly agree/agree	2.22 (1.10, 4.51)
Not sure	1.47 (0.65, 3.31)
Disagree/strongly disagree	1.00
"I see no difference between "certification" and "specialisation" in physiotherapy"	
Strongly agree/agree	0.34 (0.14, 0.83)
Not sure	0.41 (0.16, 1.02)
Disagree/strongly disagree	1.00
**Predicting interest in being a PT specializing in rheumatology **(Max-rescaled R^2 ^= 0.14, c = 0.70)	
Current arthritis caseload	
≥ 40% of patients with OA or RA	3.27 (1.69, 6.33)
<40% of patients with OA or RA	1.00
"PT Practitioners should be allowed to adjust medications and order investigative tests under the supervision of a physician"	
Strongly agree/agree	2.23 (1.02, 4.88)
Not sure	1.16 (0.46, 2.98)
Disagree/strongly disagree	1.00
"In Canada, less than 20% of PTs in arthritis rehabilitation will be interested in being a specialist"	
Strongly agree/agree	0.27 (0.10, 0.78)
Not sure	0.60 (0.28, 1.29)
Disagree/strongly disagree	1.00
**Predicting interest in being a PT Practitioner in rheumatology **(Max-rescaled R^2 ^= 0.12, c = 0.69)	
"All patients referred to see an orthopaedic surgeon for consultation should be first triaged by a PT with advanced arthritis training"	
Strongly agree/agree	5.02 (1.65, 15.34)
Not sure	3.50 (1.10, 11.09)
Disagree/strongly disagree	1.00
"All PTs working in rheumatology should be certified as Arthritis/Rheumatology Therapists"	
Strongly agree/agree	1.40 (0.71, 2.77)
Not sure	0.32 (0.11, 0.90)
Disagree/strongly disagree	1.00

PT = physiotherapist; OA = osteoarthritis; RA = rheumatoid arthritis; CI = confidence interval

The model predicting interest in being a PT specializing in rheumatology retained three statistically significant explanatory variables: 1.) current OA/RA caseload ≥ 40% (OR = 3.27, 95% CI: 1.69, 6.33); 2.) agreement to "PT Practitioners should be allowed to adjust medications and order investigative tests under the supervision of a physician" (strongly agree/agree vs. disagree/strongly disagree OR = 2.23, 95% CI: 1.02, 4.88); and, 3.) agreement to "In Canada, less than 20% of PTs in arthritis rehabilitation will be interested in being a Specialist" (strongly agree/agree vs. disagree/strongly disagree OR = 0.27, 95% CI: 0.10, 0.78). Again, all three variables' effects are monotonic (considering the ORs for not sure vs. disagree/strongly disagree) and in the expected direction in affecting the interest in being a PT specializing in rheumatology.

Finally, the model predicting interest in being a PT Practitioner in rheumatology retained two statistically significant explanatory variables. The OR for agreement to "all patients referred to see an orthopaedic surgeon for consultation should be first triaged by a PT with advanced arthritis training" is, for strongly agree/agree vs. disagree/strongly disagree, 5.02 (95% CI: 1.65, 15.34). The effect of this variable is monotonic (considering the OR for not sure vs. disagree/strongly disagree) and in the expected direction. The second variable, however, is more complicated. The OR for rating "not sure" about "all PTs working in rheumatology should be certified as arthritis/rheumatology therapists" is 0.32 (95% CI: 0.11, 0.90) when compared to the "strongly disagree/disagree" category. However, the comparison between the categories "strongly agree/agree" and "strongly disagree/disagree" failed to demonstrate a statistically significant difference (OR = 1.40, 95% CI: 0.71, 2.77).

The fit was reasonable for all three models, with c ranging from 0.69 to 0.73 versus 0.50 for a random model. R^2 ^ranged from 0.12 to 0.19, which is not unreasonable for a dichotomous model designed to contain the strongest individual predictors rather than optimize overall prediction.

## Discussion

This survey revealed a few trends that are important for planning resources to support extended scope physiotherapy practice in arthritis care. First, the vast majority of orthopaedic PTs in Canada agree that PTs play an important role in screening and the early identification of arthritis and about one in four therapists are interested in pursuing certification, specialisation or being an advanced practice PT. As there are more than 7,000 PTs practicing in orthopaedics across the country, our findings suggest that there will be sufficient therapists interested in pursuing additional training to expand their roles should suitable programs be developed. To our knowledge, this is the first study that provides evidence about the interest of PTs in pursuing additional credentials in rheumatology.

Our results also highlight a few factors that are associated with PTs' interests in pursuing extended scope practice roles, such as a high arthritis caseload, having a positive attitude toward advanced clinical roles in arthritis care and toward the formal credentialing process, and recognizing the difference between certification and specialisation. It should be noted that the regression models were exploratory in nature rather than developing prediction rules to determine who will, or will not, be interested in being an extended scope practitioner. The initial pool of independent variables was selected largely based on the researchers' clinical experience and academic knowledge in advanced physiotherapy practice; hence, it might not be exhaustive. Nonetheless, our findings have implications for regions and facilities that are planning to implement extended scope physiotherapy services, particularly in caseload planning. In addition, increasing the availability of information about existing models of care involving extended scope practitioners and their effectiveness in arthritis care may help to shape PTs' attitudes toward the new roles. Several recent reports and publications are useful for this purpose [34-36].

A key element to the implementation of advanced physiotherapy practice roles is the interest of PTs in pursuing a career in rheumatology. To this end, exposure to rheumatology theory and clinical placement during the entry-level education is a crucial step to introduce health professionals to the field, as demonstrated in other clinical areas [37]. Adequate entry-level rheumatology training is also important for PTs working in orthopedics as they are likely to see patients with OA or other types of arthritis. Previous studies on rheumatology education were conducted from the perspectives of those who designed or delivered the academic programs. However, instructors might not fully understand whether students felt the content was properly covered. By surveying the PTs directly, our study adds to the knowledge about how rheumatology was taught based on their own experience and perception.

A few gaps in entry-level rheumatology education were identified, including the coverage of assessment of assistive devices and orthoses, interpretation of basic investigative tests, and use of community and professional resources for arthritis management. Since some topics are more relevant than others to the physiotherapy practice, it is reasonable to expect less emphasis on investigative tests. However, it is disconcerting that less than 20% of PTs were satisfied with the coverage of community resources since self-management is a major component in arthritis care.

One of the challenges faced by entry-level physiotherapy programs is the limited amount of time allocated for rheumatology. Previous studies from the UK, Canada and the USA have raised the issue about insufficient instructional hours and clinical experience during entry-level education [38-40]. A survey of Canadian universities in 1997 found that the average rheumatology instructional time in undergraduate physiotherapy programs were 22.5 hours (range: 8–52 hours) [39], and only 77% offered rheumatology clinical placements. However, with the transition from a four-year baccalaureate program to a two-year master's program in most Canadian universities, the time assigned to the rheumatology component is reduced in most programs.

The gaps in entry-level training are alarming as the entry-level knowledge provides the basis for therapists to build their advanced practice skills. It should be noted that individuals in this study might have rated the coverage of a topic based on the need to use that knowledge in their current practice. Hence, they might have rated a skill that they rarely used (e.g., assessment for juvenile idiopathic arthritis) as 'adequately covered' even if it was only taught briefly during the entry-level training. Also, since this survey focuses on non-pharmacological treatments, it is unclear if medication management was adequately taught. We, however, anticipate some deficits in the coverage of this topic based on Almeida et al's finding that only 50% of physiotherapy students received moderate or in-depth classroom teaching in medication management [40].

There are several potential limitations in this study. First, the return rate of this survey is low (47.7%); hence the findings may be subjected to response bias where those who responded may be systematically different from those did not, affecting generalizability. Second, one of the professional colleges did not participate in this survey; hence our findings may not be generalizable to PTs practicing in that region. Third, we surveyed practicing PTs about their perceptions of the entry-level rheumatology education, but since more than 60% of participants graduated more than 10 year ago, their experience might not reflect that of the current physiotherapy students. Finally, results of the content rigour of entry-level education should be interpreted with caution since the measurement properties of the scale have not been tested.

Despite the limitations, this survey provides a comprehensive view on the interests and readiness of Canadian PTs to embark on extended scope practice in rheumatology. Furthermore, we were able to capture the opinions of both English-speaking and French-speaking health professionals by providing the questionnaire in both official languages. The rigorous translation protocol we employed has ensured that both versions were equal and understandable to the participants.

## Conclusion

This study demonstrated the interests of PTs in pursuing certification, specialisation, and extended scope practice roles, and highlighted the needs to improve entry-level rheumatology curricula in order to prepare PTs to pursue more advanced training. One solution to improve PTs' training is to develop standards for entry-level rheumatology curricula, such as those established for PTs, occupational therapists and nurses in the UK [41], and for medical trainees in Canada [42]. Furthermore, since other health professionals such as nurses, occupational therapists and pharmacists are also expanding their roles to fill the gaps in arthritis management [36], a similar survey can provide information for guiding the development of standards for training and credentialing. Finally, since most of the previous research evaluated the effect of extended scope PTs on waitlist management and patient satisfaction, future research should also focus on assessing patient outcomes.

## Competing interests

The authors declare that they have no competing interests.

## Authors' contributions

LCL, MDW, ES, and MT developed the study protocol. LCL, MDW, ES, MT, and LC developed the survey instrument and conducted the survey. LCL and ECS participated in the data analysis. LCL drafted the manuscript. All authors provided comments and approved the final version.

## Pre-publication history

The pre-publication history for this paper can be accessed here:


